# Ion carrier modulated MRI contrast†

**DOI:** 10.1039/d3sc03466f

**Published:** 2024-07-18

**Authors:** Anna M. Duncan, Connor M. Ellis, Hannah Levingston, Aidan Kerckhoffs, Ferenc E. Mózes, Matthew J. Langton, Jason J. Davis

**Affiliations:** a Department of Chemistry, University of Oxford South Parks Road Oxford OX1 3QZ UK jason.davis@chem.ox.ac.uk +44(0)1865272690 +44(0)1865275914; b Oxford Centre for Clinical Magnetic Resonance Research, Radcliffe Department of Medicine, University of Oxford, Level 0, John Radcliffe Hospital Oxford OX3 9DU UK

## Abstract

An ion-responsive MRI contrast agent based on a POPC liposomal scaffold is generated that displays a large amplitude relaxivity switch. Entrapment of MR active Gd-DOTA within cholesterol-doped, *i.e.*, membrane rigidified, liposomes dampens the MR response through diminished water exchange across the lipid bilayer. Relaxivity is re-established by integration of ion carriers in the liposome membrane to mediate solvated ion flux.

## Abstract 2

The activated specific transmembrane symport of cations/anions and associated water provides a mechanism by which a significant switch in MRI contrast/relaxivity can be generated with paramagnetically loaded liposomes.

## Graphical abstract

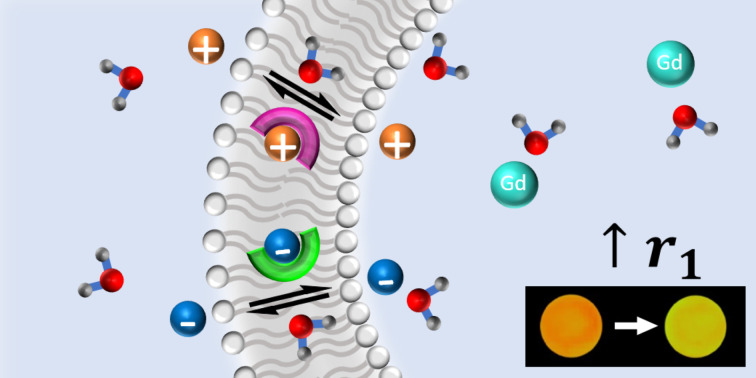

## Introduction

Magnetic contrast agents, capable of enhancing image sensitivity within a MRI scan, are central to the effective diagnosis (and consequent prognosis) of a very broad range of clinical conditions and disease states.^1^ Nanoparticulate contrast agents (NP CAs) offer enhanced signal-to-noise ratios and high associated relaxivity over their molecular counterparts, enriching MR image acquisition, often with accompanying improved circulation times and lower doses (and thus potential for reduced toxicity).^2^ Examples are rich in number and chemical flavour, being based on (super)paramagnetically doped scaffolds that are largely organic, exclusively inorganic, or hybrid in nature.^3–5^ In heralding a new era of disease specific reporting, many of these are able to respond to a desired environmental stimulus (such as pH, ions, or biomolecules).^6^ Paramagnetic liposomes are a potentially potent subclass of these, possessing high biocompatibility, and an associated capacity for payload incorporation within the interior cavity.^7^

Liposomal CAs have been synthesised either through the immobilisation of paramagnetic probes on the membrane surface, or by encapsulation of magnetic probes such as Gd-chelates within the intra-vesicular volume of the phospholipid bilayer. These approaches have supported the incorporation of a high associated payload of 10^4^ to 10^5^ molecular chelates,^8^ such as gadoteric acid (Gd-DOTA), that can promote *T*_1_ contrast generation *in vivo*.^9^ A number of thermosensitive DPPC liposomes capable of supporting thermally activated *T*_2_ relaxivity have also been reported.^9,10^ In addition to these, iron oxide nanoparticles (IONPs) have been integrated within the intra-vesicle space facilitating *T*_2_ contrast.^11–13^ The influence of water exchange (limited and/or thermally activated) across the lipid bilayer is highlighted in this prior work; contrast is limited by membrane water permittivity and, thus, potentially responsive to any bilayer fragmentation or change in rigidity (thermally or chemically imposed, such as through the integration of cholesterol).^14,15^ Where DPPC liposomes have been loaded with both a therapeutic agent (Camptosar) and IONPs, thermally induced irreversible fragmentation enables both agent delivery and direct monitoring of this by *T*_2_ weighted MRI.^16^

Ion channel mis-regulation is directly causative of several diseases; an ability to monitor exogeneous ion imbalance *in vivo* would be beneficial for enhancing our understanding of these processes.^17^ Ion carriers capable of mediating transmembrane transport may offer valuable therapeutic intervention,^18,19^ and their development is an area of intense interest. Numerous synthetic ion transporters are known, including some that exhibit stimulus responsive behaviour.^20^ Since ions are heavily solvated in aqueous solution,^21^ we hypothesised that recognition mediated transport would be accompanied by at least some associated exchange of water across the bilayer. We expected that this, in turn, would support a mechanistically novel platform for which image contrast switches in response to exogenous ions.

## Results and discussion

MR active 1-palmitoyl-2-oleoyl-*sn*-glycero-3-phosphocholine (POPC) liposomes were here generated by a typical extrusion process, using a polycarbonate membrane with a pore size of 100 nm. POPC lipid membranes with incorporated cholesterol are highly biologically relevant, as they represent an idealised and highly tuneable model of the biomembrane.^22^ All liposomal samples exhibited high levels of colloidal stability (PDI < 0.3 for all samples), with an associated hydrodynamic size of 109.50 ± 4.51 nm and *ζ*-potential of −6.94 ± 0.71 mV for non-cholesterol modified POPC liposomes (POPC–C_0%_–N) measured immediately after synthesis (as resolved by dynamic light scattering (DLS) measurements, ESI 9(a)†). Gd-DOTA was incorporated within the intra-vesicular cavity at the point of liposome formation, by dispersing the desired concentration of chelate in water and adding to the dried lipid film (ESI†). Resuspension, freeze-thawing and subsequent extrusion steps were conducted to afford the desired MR active vesicles (see the ESI† for procedure details). The liposomes were shown to be stable for 72 h, sufficient for MR analysis, with no significant differences in size or surface charge (ESI 9(a) and 9(b)†), observations of translational importance.^23^ A range of Gd-DOTA loading densities were investigated (ESI 10†), with 75 mM ultimately added at the point of synthesis, to bias water exchange as rate limiting in relaxivity/contrast. These liposomes exhibited longitudinal relaxivity values (*r*_1_ = 4.70 ± 0.02 mM^−1^ s^−1^ at 1.41 T and 298 K, in water) characteristically indicative of effective diffusive water access across the lipid membrane. Notably, this water flux could be controllably “tuned down” significantly (at 1.41 T, associated *r*_1_ = 2.52 ± 0.09 mM^−1^ s^−1^, ESI 11†) by doping the bilayer membrane with 45 mol% cholesterol, (POPC–C_45%_–N), a process associated with a negligible change in hydrodynamic size (99.35 ± 3.87 nm) and *ζ*-potential (−9.93 ± 1.12 mV) with a transmission electron microscopy (TEM) resolved diameter equal to 98.2 nm (ESI 12†). Good colloidal stability over 90 hours was also observed upon incubation of POPC–C_45%_–N and POPC–C_45%_–T in serum (ESI 13†) with no significant variation in number mean. Osmotic shock experiments using DLS additionally confirmed the role of cholesterol in gating water flux (ESI 14†), whereby liposomes composed solely of POPC exhibited a ∼20% reduction in the hydrodynamic diameter between incubation in pure water and 20 mM KCl solution. In comparison, liposomes doped with a high concentration of cholesterol (*i.e*., >40%)^24^ displayed no significant variation in size when subjected to the same conditions, confirming bilayer rigidification.

It is known that gramicidin-A (GramA) peptide channels can be reliably integrated into lipid bilayers, including those presented by liposomes, and are able to mediate water flux by a Grotthuss hopping mechanism at rates exceeding 10^−6^ cm^−2^ s^−1^ (Fig. 2(a)).^25,26^ The integration of GramA (2 mol% w.r.t. POPC) within the cholesterol modified liposomes was observed to support a very significant (>30%) boost in MR relaxivity for liposomes possessing 45–50 mol% cholesterol loading (Fig. 2(b)). Significantly, these experiments demonstrate that initially low liposomal contrast could be switched on if water flux across the bilayer is promoted.

**Fig. 1:**
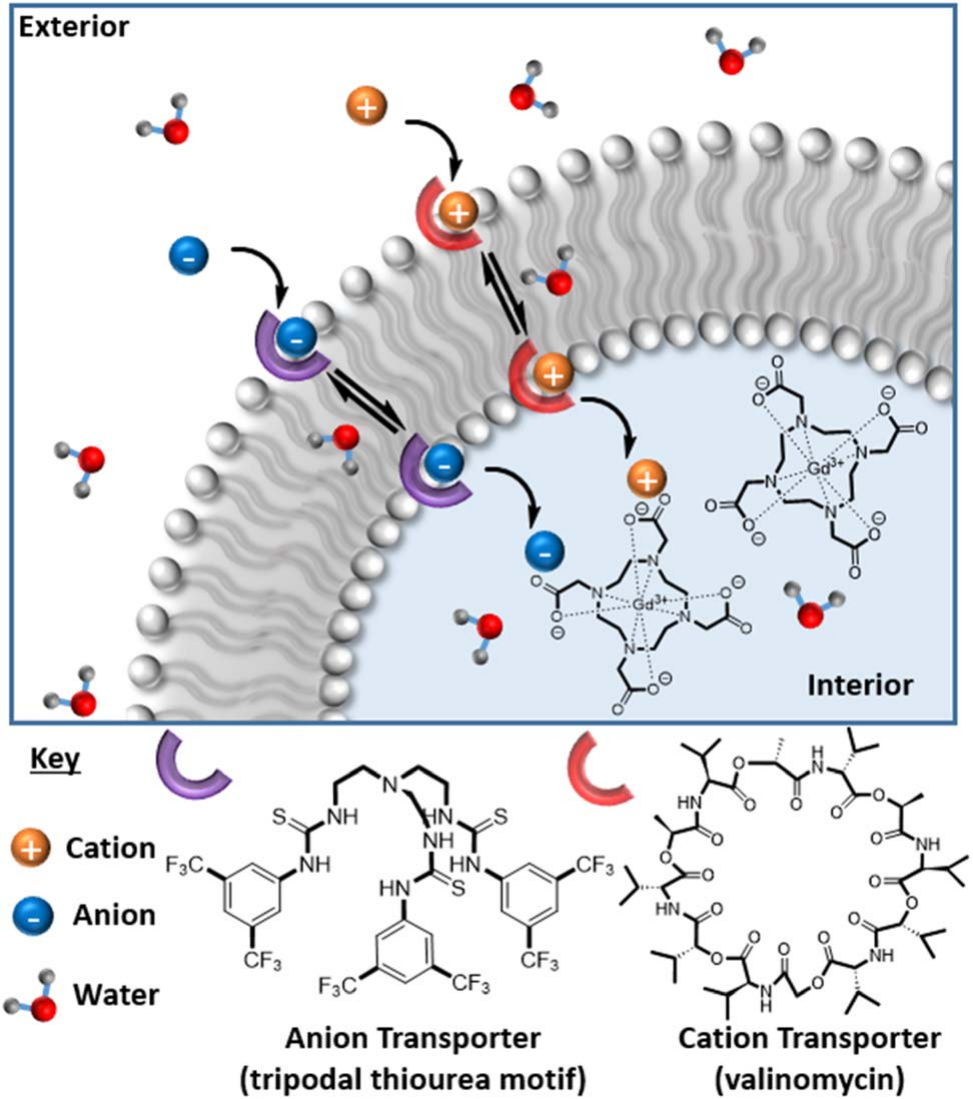
A schematic illustrating the transfer of electrostatically bound K^+^ to Vln and Cl^−^ by tripodal thiourea across the lipid bilayer of MR-active liposomes, accompanied by associated water transport which switches on MR image contrast.

We then sought to control (ion associated) water flux by integration of selective ion carriers within the liposomal membrane. Valinomycin (Vln) is an antibiotic of natural origin and an effective K^+^ transmembrane transporter with rates in the region of 10^4^ s^−1^ (Fig. 1).^27–30^ This K^+^ ionophore, along with a tripodal thiourea anionophore developed by Busschaert *et al.* capable of efficiently transporting Cl^−^,^31^ were co-incorporated into the membrane of cholesterol-modified lipid vesicles (POPC–C_45%_–T) by addition of a 1 mol% (w.r.t. POPC) DMSO stock solution to the liposome suspension. Extensive prior work supports non-destructive transporter integration by this method.^19^ Some water transport is known to be associated with the resultant ion transfer across the lipid bilayer.^32^ The rate of diffusion of water across an unmodified lipid membrane has been theoretically estimated to be 2 × 10^−4^ to 50 × 10^−4^ cm s^−1^ and is greatly reduced upon incorporation of cholesterol into the membrane (confirmed using stopped flow osmotic analyses).^33,34^ Given that the associated ion transport rates for the K^+^ and Cl^−^ transporters used in this work are in the region of 10^2^ to 10^4^ s^−1^, we anticipated a marked increase in water flux in their presence.^27,31,32^ Osmotic analyses herein confirm the coupling of anion/cation symport exchange across the bilayer to an enhanced water flux (see ESI 16†). Carrier mediated KCl symport (co-transport) and the associated increased water flux specifically correlate with an observable hydrodynamic size modulation as resolved by DLS (ESI 15 and 17†). Real time analyses of this (ESI 18†) enable an estimation of some 750% increase in net water flux when both mobile carriers are present (ESI 19†). Pleasingly, this carrier mediated transmembrane water flux leads to substantial (60%) relaxivity change; notably this is observed only in the dual integration of both transporters and in the presence of physiologically relevant levels of KCl *i.e.*, through the activation of net neutral K^+^/Cl^−^ symport transport.^35^ Integration of either the cation or anion transporter alone in the presence of the same levels of KCl results in no detectable change in relaxivity (ESI 20(a) and (b)†). The requirement for KCl symport was also confirmed through ion transport experiments in liposomes using a fluorescence assay, which demonstrated that efficient Cl^−^ net flux in the presence of K^+^ requires both the cationophore and the anionophore to be present (ESI 21†).^36^

**Fig. 2:**
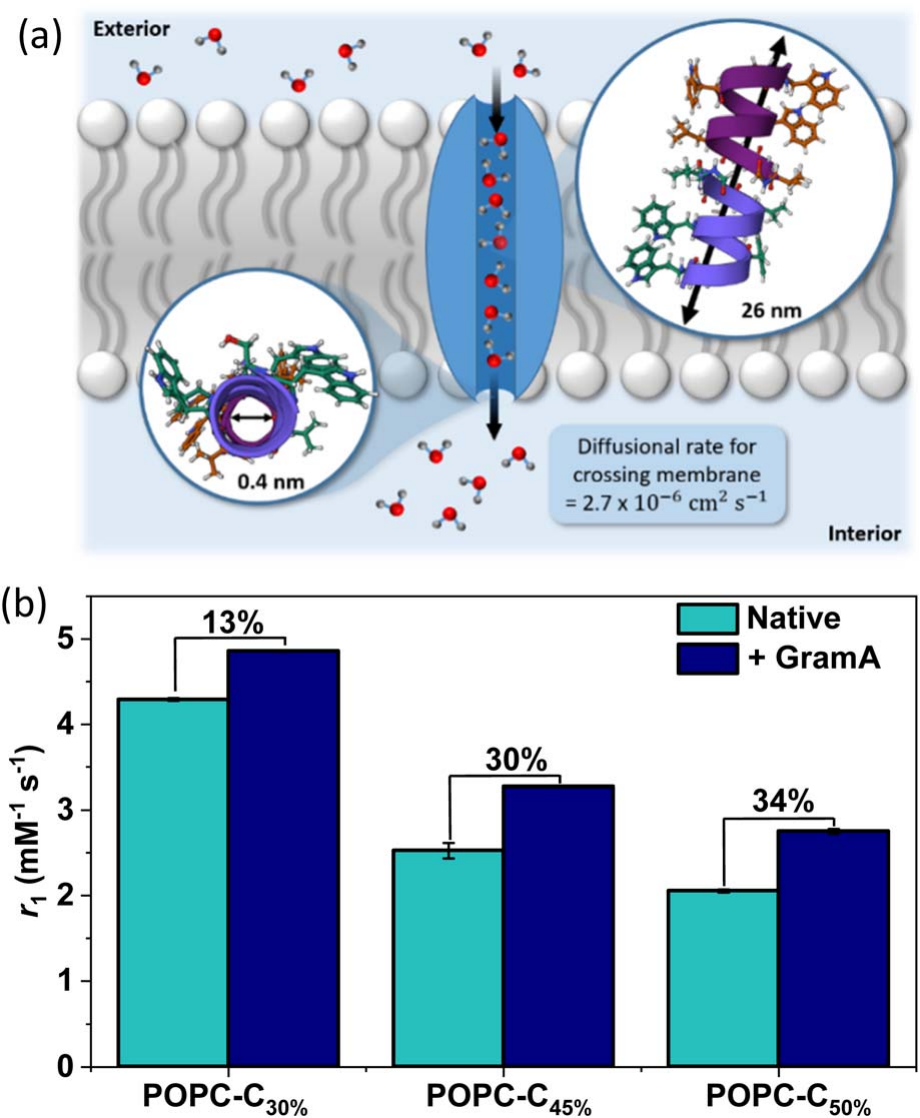
(a) A schematic highlighting the transport of water molecules from the exterior solution to the interior pool of the liposomes, mediated by GramA (PDB ID: 1MAG).^26^ The pore diameter and the length of GramA are 0.4 nm and 26 nm respectively, with the diffusion rate of water highlighted.^25,26^ The diameter of the pore channel is narrow and therefore it enables a single chain of 7–9 water molecules to align within it.^25^ (b) The influence of GramA integration (2 mol% w.r.t. POPC) on the relaxivity (obtained at 1.41 T, 298 K) of pre-formed Gd loaded vesicles, across a range of cholesterol doping levels, to highlight the increased rigidification at higher cholesterol loading. Error bars for all data points were obtained from a linear regression analysis on linear plots of 1/*T*_1_*versus* [Gd(iii)] to obtain a value for the gradient, which is equal to *r*_1_ ± 1 s d.

**Fig. 3:**
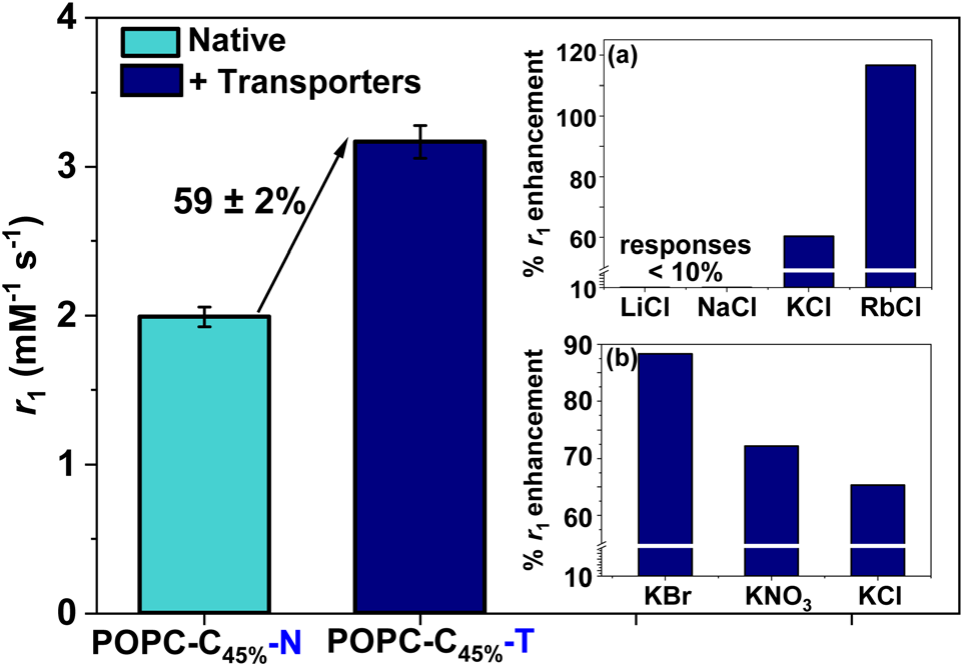
Longitudinal relaxivity values obtained at 1.41 T, 298 K for 45 mol% cholesterol-doped liposomes in both the absence and presence of KCl (1.08 mM) and transporters (1 mol% each). Error bars represent 1 s d. on either side of the mean *r*_1_ value across two independent samples. Inset (a) shows the cation selectivity (1.08 mM MCl) with the greatest response observed for RbCl (∼120%). Analogous Li and Na responses were <10%. Inset (b) displays the anion selectivity trend (1.08 mM KX).

**Fig. 4:**
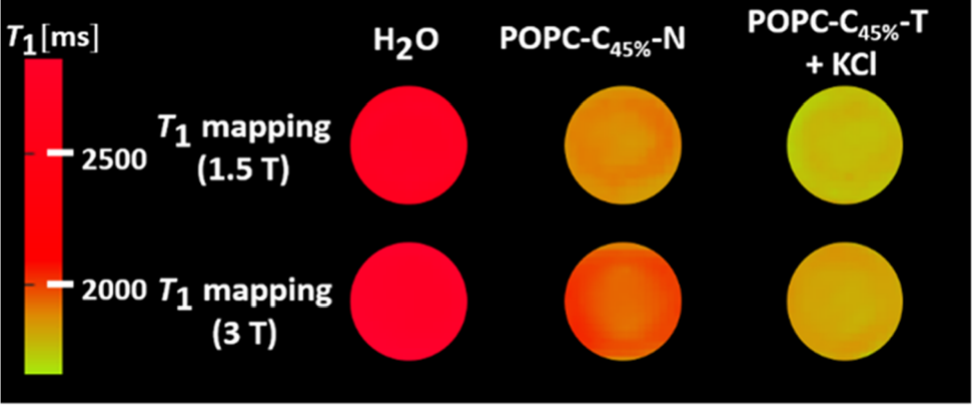
*T*_1_ weighted MRI maps, recorded at 1.5 T and 3 T (298 K) for the cholesterol doped lipids in the absence and presence of the transporters and KCl salt (1.08 mM).

*T*_1_ was independent of the KCl concentration across the physiological range (0.1–5.0 mM), consistent with the high activity of both ionophores (see ESI 22,† measured at 1.4 T).^27,31,37^ Assessing the anion selectivity of this we noted relative enhancements in *r*_1_ of 83% and 67% were observed experimentally in the presence of 1.08 mM Br^−^ and NO_3_^−^ respectively (see Fig. 3(b)), seemingly correlating with the ease of anion desolvation and hence transport efficiency.^31^ Cation selectivity trends were entirely consistent with the known Vln binding selectivities (see Fig. 3(a)).^38^ Since molecular Gd chelates are used in approximately 40% of all MRI examinations,^39^ we then demonstrated that this ion transport could generate significant image contrast change within *T*_1_ weighted images obtained on a clinical MRI scanner (at 1.5 T and 3 T, at 298 K; Fig. 4). *T*_1_ itself predictably increases for both POPC–C_45%_–N and POPC–C_45%_–T liposomes as magnetic field strength (*B*) increases (ESI 23†).

**Fig. 5:**
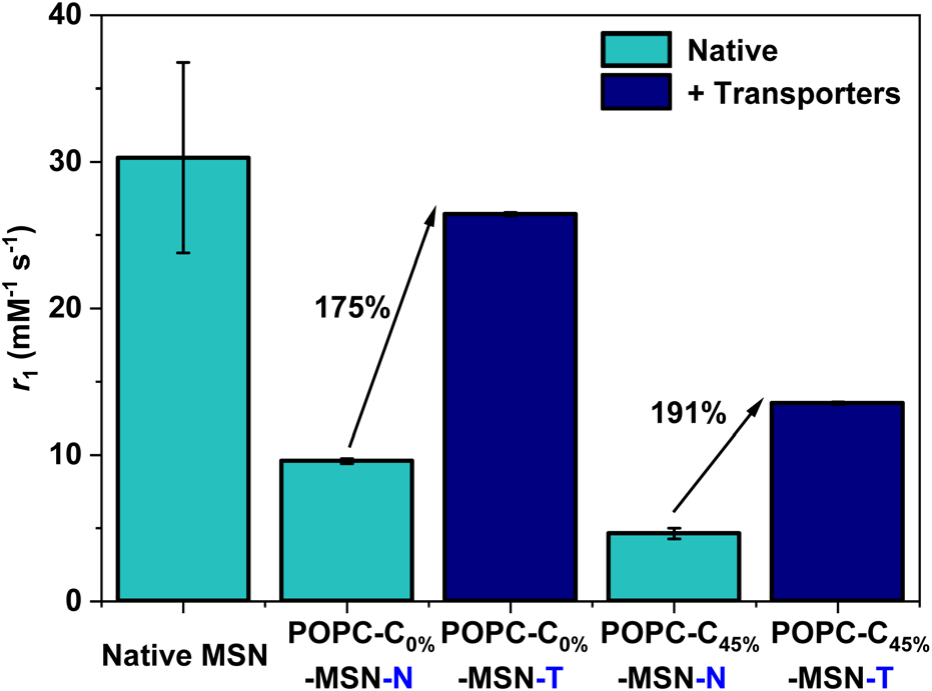
*r*_1_ values obtained at 1.41 T, 298 K for the POPC–MSNs (for 0 mol% and 45 mol% cholesterol modified bilayers), in both the absence and presence of the ionophores (1 mol% each), with the associated relative percentage switch in *r*_1_ highlighted. Error bars are calculated as described in Fig. 2(b).

Building on the observations of ion flux mediated contrast switching with Gd-DOTA carrying liposomes, we integrated Gd-doped mesoporous silica nanoparticles (Gd-MSNs) into functional POPC liposomes (POPC–C_45%_–MSN–N) through fusion (ESI 7 and 8 for characterisation, and ESI 24–27†).^40^ Characterisation by TEM and DLS confirms that these Gd-MSNs are consistently and effectively encapsulated within lipid bilayers, as expected.^41,42^ Since paramagnetic MSNs support much higher levels of relaxivity than molecular chelates,^4^ we hypothesied that the initial water transmembrane transport (and the small internal water pool) would be more limiting. Pleasingly, ion transport MR relaxivity turn-on is not only fully consistent with that observed with Gd-DOTA, but also indeed of a greater turn on magnitude; for both 0 and 45 mol% cholesterol doped lipid bilayers, relaxivity switches close to 200% were observed (Fig. 5) with Gd-MSN doped liposomes containing 1 mol% of both cation and anion transporters (POPC–C_45%_–MSN–T) in the presence of a 1.08 mM KCl gradient.

## Conclusions

This work, then, demonstrates that the general principal of ion mediated water flux across the membrane of MR active liposomes is associated with significant modulation of MR relaxivity and associated image contrast. A “switch off” of paramagnetic liposome MR contrast can be established through lipid membrane rigidification and selectively re-established through water associated ion transport using synthetic ionophores. This proof of concept lays the groundwork for potentially imaging ion imbalance and the associated diseases thereof. Derivations which are both ratiometric (by, for example, integrating a cholesterol-anchored ^19^F probe)^43^ and enzyme activated^44^ can be envisaged.

## Author contributions

C. M. E., A. M. D., and H. L., designed the liposomes and acquired the experimental data. A. K. designed the liposomes and synthesised the tripodal thiourea anionophore. F. E. M. acquired the MRI data. M. J. L. and J. J. D. conceptualised and designed the project. C. M. E., A. M. D., M. J. L. and J. J. D. wrote the manuscript, which was approved by all authors.

## Conflicts of interest

There are no conflicts to declare.

## Supplementary Material

SC-015-D3SC03466F-s001

## Data Availability

The data supporting this article have been included as part of the ESI.†

## References

[cit1] Wahsner J., Gale E. M., Rodríguez-Rodríguez A., Caravan P. (2019). Chem. Rev..

[cit2] Pellico J., Ellis C. M., Davis J. J. (2019). Contrast Media Mol. Imaging.

[cit3] Ni D., Bu W., Ehlerding E. B., Cai W., Shi J. (2017). Chem. Soc. Rev..

[cit4] Yuan D., Ellis C. M., Davis J. J. (2020). Materials (Basel).

[cit5] Zhang W., Liu L., Chen H., Hu K., Delahunty I., Gao S., Xie J. (2018). Theranostics.

[cit6] Ellis C. M., Pellico J., Davis J. J. (2019). Materials.

[cit7] Xia Y., Xu C., Zhang X., Ning P., Wang Z., Tian J., Chen X. (2019). Nanoscale.

[cit8] Ayyagari A. L., Zhang X., Ghaghada K. B., Annapragada A., Hu X., Bellamkonda R. V. (2006). Magn. Reson. Med..

[cit9] Fossheim S. L., Fahlvik A. K., Klaveness J., Muller R. N. (1999). Magn. Reson. Imaging.

[cit10] Yeo S. Y., De Smet M., Langereis S., Vander Elst L., Muller R. N., Grüll H. (2014). Biochim. Biophys. Acta – Biomembr..

[cit11] German S. V., Navolokin N. A., Kuznetsova N. R., Zuev V. V., Inozemtseva O. A., Anis’kov A. A., Volkova E. K., Bucharskaya A. B., Maslyakova G. N., Fakhrullin R. F., Terentyuk G. S., Vodovozova E. L., Gorin D. A. (2015). Colloids Surf..

[cit12] Meincke M., Schlorf T., Kossel E., Jansen O., Glueer C.-C., Mentlein R. (2008). Front. Biosci..

[cit13] Béalle G., Di Corato R., Kolosnjaj-Tabi J., Dupuis V., Clément O., Gazeau F., Wilhelm C., Ménager C. (2012). Langmuir.

[cit14] Jain M. K., Touissaint D. G., Cordes E. H. (1973). J. Membr. Biol..

[cit15] Bittman R., Blau L. (1972). Biochemistry.

[cit16] Lu Y. J., Chuang E. Y., Cheng Y. H., Anilkumar T. S., Chen H. A., Chen J. P. (2019). Chem. Eng. J..

[cit17] Langton M. J., Serpell C. J., Beer P. D. (2016). Angew. Chem., Int. Ed..

[cit18] Gale P. A., Davis J. T., Quesada R. (2017). Chem. Soc. Rev..

[cit19] Bickerton L. E., Johnson T. G., Kerckhoffs A., Langton M. J. (2021). Chem. Sci..

[cit20] Langton M. J. (2021). Nat. Rev. Chem..

[cit21] Bieske E. J. (2003). Chem. Soc. Rev..

[cit22] Pillai V. V. S., Kumari P., Benedetto A., Gobbo D., Ballone P. (2022). J. Phys. Chem. B.

[cit23] Schubert J., Chanana M. (2018). Curr. Med. Chem..

[cit24] Briuglia M.-L., Rotella C., McFarlane A., Lamprou D. A. (2015). Drug Deliv. Transl. Res..

[cit25] Chiu S. W., Subramaniam S., Jakobsson E. (1999). Biophys. J..

[cit26] Ketchem R. R. , Lee K.-C., Huo S. and Cross T. A., J. Biol. NMR, 1996, 8, 1 14, PDB ID: 1MAG, 10.1007/BF01881753 8810522

[cit27] Stark G., Ketterer B., Benz R., Läuger P. (1971). Biophys. J..

[cit28] Berezin S. K. (2015). J. Membr. Biol..

[cit29] Casnati A., Pochini A., Ungaro R., Bocchi C., Ugozzoli F., Egberink R. J. M., Struijk H., Lugtenberg R., De Jong F., Reinhoudt D. N. (1996). J. Am. Chem. Soc..

[cit30] Gresh N., Etchebest C., de la Luz Rojas O., Pullman A. (1981). Int. J. Quant. Chem..

[cit31] Busschaert N., Wenzel M., Light M. E., Iglesias-Hernández P., Pérez-Tomás R., Gale P. A. (2011). J. Am. Chem. Soc..

[cit32] Levitt D. G., Elias S. R., Hautman J. M. (1978). Biochim. Biophys. Acta – Biomembr..

[cit33] Haines T. H. (1994). FEBS Lett..

[cit34] Mathai J. C., Tristram-Nagle S., Nagle J. F., Zeidel M. L. (2008). J. Gen. Physiol..

[cit35] Zacchia M., Abategiovanni M. L., Stratigis S., Capasso G. (2016). Kidney Dis..

[cit36] Chvojka M., Singh A., Cataldo A., Torres-Huerta A., Konopka M., Šindelář V., Valkenier H. (2024). Analysis Sensing.

[cit37] Hoss S., Elizur Y., Luria D., Keren A., Lotan C., Gotsman I. (2016). Am. J. Cardiol..

[cit38] Su Z., Mrdenovic D., Sek S., Lipkowski J. (2020). J. Solid State Electrochem..

[cit39] Wahsner J., Gale E. M., Rodríguez-Rodríguez A., Caravan P. (2019). Chem. Rev..

[cit40] Mornet S., Lambert O., Duguet E., Brisson A. (2005). Nano Lett..

[cit41] Butler K. S., Durfee P. N., Theron C., Ashley C. E., Carnes E. C., Jeffrey Brinker C. (2016). Small.

[cit42] Durfee P. N., Lin Y.-S., Dunphy D. R., Muñiz A. J., Butler K. S., Humphrey K. R., Lokke A. J., Agola J. O., Chou S. S., Chen I.-M., Wharton W., Townson J. L., Willman C. L., Jeffrey Brinker C. (2016). ACS Nano.

[cit43] Zhu J., Zhang H., Chen K., Li Y., Yang Z., Chen S., Zheng X., Zhou X., Jiang Z.-X. (2020). Adv. Healthcare Mater..

[cit44] Ahmad M., Johnson T. G., Flerin M., Duarte F., Langton M. J. (2024). Angew. Chem., Int. Ed..

