# Supplementary material for: Ion carrier modulated MRI contrast
Source: Chem Sci. 2024 Jul 18;15(34):13937–41. doi: 10.1039/d3sc03466f (PMC11310829; doi:10.1039/d3sc03466f)
Supplement: SC-015-D3SC03466F-s001 [file SC-015-D3SC03466F-s001.pdf]

Electronic Supplementary Material (ESI) for Chemical Science.
This journal is © The Royal Society of Chemistry 2024

Supplementary Information:

Experimental section:

Chemicals:

Phosphatidylcholine (POPC) was purchased from Avanti Polar Lipids, Inc. Gadolinium (III) 1,4,7,10–tetraazacyclododecane–1,4,7,10–tetraacetate (Gd-DOTA) was purchased from Macrocyclics™. All other chemicals were purchased from Sigma-Aldrich and used as received. Ultrapure water used for all solutions was obtained from the Milli-Q Academic Millipore system (with a resistivity of 18.2 MΩ cm). Serum (human male AB plasma) was purchased from Sigma-Aldrich, U.K., and is of USA origin. The serum was sterile-filtered and prepared in 1.08 mM KCl (aq) at concentrations of 2%, 5% and 50% respectively.

Synthetic scheme:

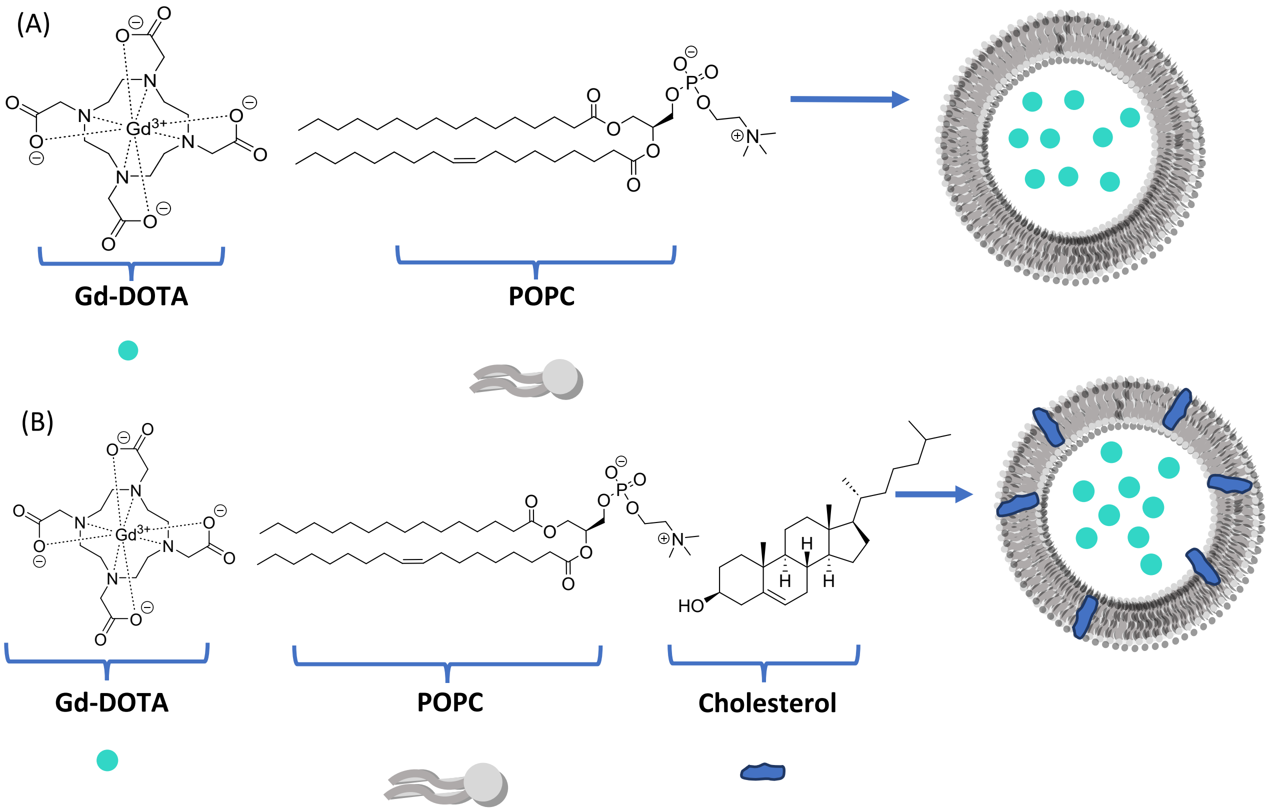

ESI 1. Synthetic scheme to generate the MRI active liposomes, employing POPC as the synthetic phospholipid building block. Gd-DOTA is encapsulated within the vesicles, enabling a MRI active capability, with varying degrees of cholesterol added (0 - 50 mol%) at the point of synthesis to control

the rigidity and hydrophobicity of the lipid bilayer. (A) Displays the non-cholesterol modified (*i.e.,* native POPC) Gd-DOTA doped liposomes whilst (B) shows those vesicles with cholesterol in the bilayer.

Abbreviations

POPC-$C_{i\%}$(-N) = Cholesterol doped (i%) paramagnetic POPC liposomes

POPC-$C_{i\%}$-T = Transporter modified cholesterol doped (i%) paramagnetic POPC liposomes

POPC-$C_{i\%}$-MSN(-N) = MSN supported cholesterol doped (i%) POPC lipid bilayer

POPC- $C_{i\%}$-MSN-T = Transporter modified MSN supported cholesterol doped (i%)

Preparation of Gd-DOTA doped cholesterol loaded POPC liposomes:

Liposomes were prepared by an extrusion process, using the Avestin "LiposoFast" extruder apparatus, and polycarbonate membranes (100 nm) in order to produce uniformly sized liposomes. Pre-formed liposomes were produced by adding a lipid stock solution in chloroform (266 µL), possessing varying cholesterol concentrations (0 – 50 mol% w.r.t POPC), to a round bottom flask. The solvent was then removed by rotary evaporation, and dried on the high-vacuum line for 8 hrs. A solution (75 mmol) of Gd-DOTA was prepared by dissolving the Gd-DOTA in ultrapure water and sonicating until fully dispersed. This solution (1 mL) was added to the flask containing the film of dried lipids, with the lipids suspended in solution by vortexing the flask for 30 seconds until a cloudy suspension was formed. The lipid suspension was then subjected to 5 freeze-thaw cycles using liquid nitrogen and a water bath (40°C) to ensure that unilamellar vesicles were formed. This solution was extruded 19 times through a polycarbonate membrane (Avestin, pore size 100 nm) at room temperature, before the extra-vesicular components were then removed by size exclusion chromatography using GE Healthcare PD-10 desalting columns prepacked with Sephadex G-25 medium using ultrapure water as the eluent.

Incorporation of ion transporters and salt solutions:

For all analyses the vesicles were split into both a control (native) sample and one to be modified with the transporters (valinomycin, Vln, and the tripodal thiourea chloride carrier), enabling a direct comparison between the two. For the relaxivity measurements three dilutions of each batch (1x, 0.5x and 0.25x), using water as the solvent, were prepared. For the vesicles possessing the transporters,

Vln (10 µL of 1.25 mM solution in DMSO, 1 mol% w.r.t. POPC), the tripodal thiourea transport motif (10 µL of 1.25 mM solution in DMSO, 1 mol% w.r.t. POPC) and MX salt solution (100 µL of 10 mM solution) were added, with the dilutions prepared in the same manner using water. For the addition of GramA, 5 µL of 5 mM DMSO stock solution was externally added to the vesicles, 2 mol% w.r.t POPC.

Formation of Double Delay Co-Condensation NH_2-MSNs

The method by which NH_2-MSNs, DOTA-MSNs and Gd-MSNs are synthesised follows that reported by Davis *et al.*.[1] These DOTA-MSNs employ low loadings of Gd and achieve high relaxivity values by tuning the sub-particle localisation of Gd(III) chelates within the MSN pore channels.[1] Cetrimonium bromide (CTAB, 0.64 g) and triethanolamine (TEOA, 1.03 g) were dissolved in a mixture of ethanol: water (EtOH:H_2O, 1.88:16.2 mL). The solution was then stirred at 80 °C for 20 min, with tetraethyl orthosilicate (TEOS, 1.454 mL) then added dropwise (at a rate of 1 mL min^{-1}). 10 min after the TEOS addition, 3-aminopropyltriethoxysilane (APTES, 2.6 µL) was added. 50 min after the addition of APTES, both APTES (2.6 µL, 0.15%) and TEOS (2.23 µL) were added to the reaction mixture. The mixture was then stirred vigorously at 80 °C for a further 60 min, resulting in a total reaction time of 2 h. The reaction mixture was then left to cool to room temperature, with the particles collected by centrifugation at 13,500 rpm for 20 min. The NH_2-MSNs were purified by resuspending the MSNs in EtOH (~ 25 mL) and collecting by centrifugation (at 13,500 rpm, 20 min) a further two times. The particles were then resuspended and subsequently left to sonicate for 30 min in acidic EtOH (10 v/v%) and collected by centrifugation (13,500 rpm, 20 min) to ensure the complete removal of the surfactant template. The MSNs underwent the resuspension and centrifugation process in EtOH a further two times, after which they were dried overnight under vacuum (T = 298 K, P = 1 – 2 mbar, Thermo Scientific™ Vacutherm Vacuum Heating and Drying Oven).

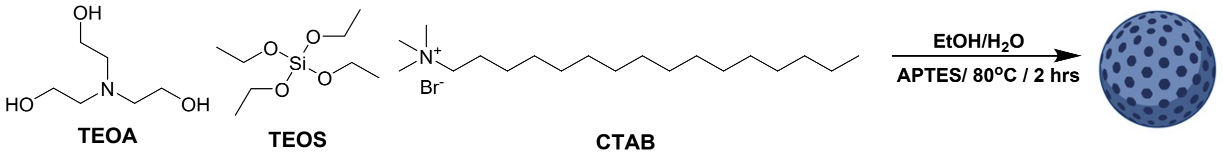

ESI 2. Synthetic scheme for the synthesis of the MSNs, employing a double delay co-condensation procedure (specifically a modification of the Stöber process). Created with BioRender.com.

Synthesis of DOTA-MSNs

NH_2-MSNs (100 mg) were dispersed in dimethylformamide (DMF, 7.5 mL) by sonication (VWR Ultrasonic Cleaner, USC-TH), to which 1,4,7,10-Tetraazacyclododecane-1,4,7,10-tetraacetic acid mono-*N*-hydroxysuccinimide ester (DOTA–NHS ester, 2.625 mmol) and triethylamine (TEA, 150 µL) were added. The solution was then left under vigorous stirring at room temperature for 24 h. The particles were collected by centrifugation (13,500 rpm, 20 min), and resuspended in EtOH (~ 25 mL). These DOTA modified MSNs were collected by centrifugation (13,500 rpm, 20 min), with the EtOH resuspension/centrifugation process completed for a total of three cycles.

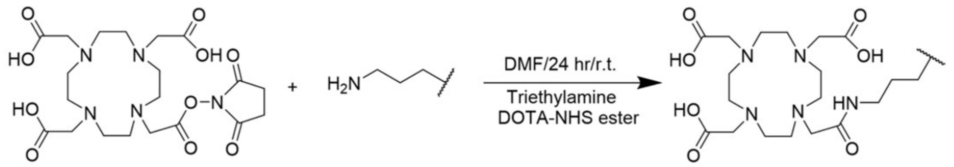

ESI 3. Reaction scheme summarising the synthesis of the DOTA-MSNs.

Synthesis of Gd-MSNs

The as-synthesised DOTA-MSNs were dispersed in EtOH (10 mL, by sonication) to which $GdCl_3.6H_2O$ (10 µmol) was added, and the solution left under vigorous stirring for 24 h at room temperature. The desired Gd-doped nanoparticles (Gd-MSNs) were collected by centrifugation (13,500 rpm, 20 min), and resuspended in EtOH (~ 25 mL). These Gd-doped MSNs were collected by centrifugation (13,500 rpm, 20 min), with the EtOH resuspension/centrifugation process completed for a total of three cycles, and the desired Gd-MSNs then left to dry under vacuum overnight.

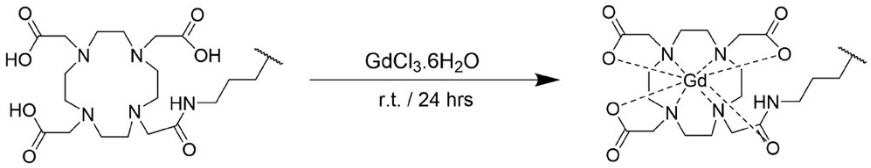

ESI 4. Reaction scheme summarising the synthesis of the Gd-MSNs.

It is known that altering the placement of Gd-DOTA, such that Gd(III) is bound at the periphery of the pore, where water exchange with bulk is most facile, promotes the highest relaxivities (33.57 ± 1.29 $mM^{-1}\ s^{-1}$ at 7 T).[1]

Gd-MSNs incorporation within the liposome

A 1 mL dispersion of the Gd-MSNs (2.5 mg mL^{-1}) in water was added to a pre-prepared liposome suspension (1 mL, prepared in PBS, otherwise the synthesis is as previously described) and sonicated for 15 s, to initiate liposomal fusion onto the silica architecture. The mixture was centrifuged at 10,000 rpm for 10 min before the supernatant (consisting of any excess unreacted liposomes) was removed, the POPC bilayer wrapped Gd-MSNs re-dispersed in phosphate buffered saline (PBS, 2 mL), and subsequently centrifuged again under the same conditions. The supernatant was removed, and the final product was redispersed in PBS (2 mL) for subsequent DLS analysis.

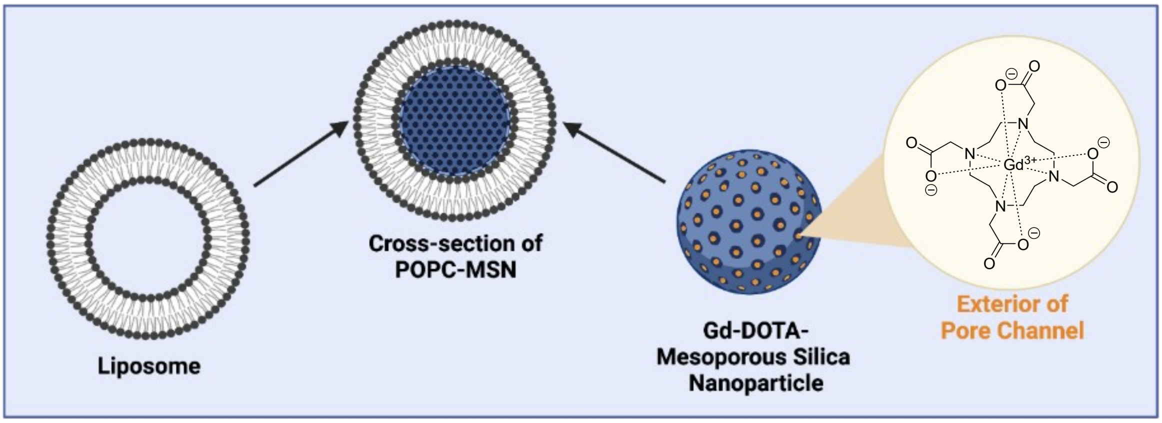

ESI 5. POPC-MSNs (or protocells) were formed from the fusion of a liposome lipid bilayer onto pre-synthesised Gd-MSNs.[2] Created with BioRender.com.

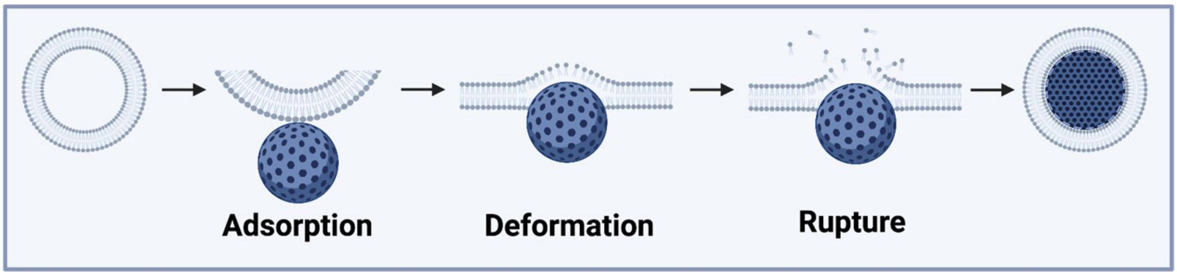

ESI 6. The fusion of a lipid bilayer onto the MSNs has been previously observed by cryo-EM to occur in the following stages: adsorption of the MSN onto the bilayer, subsequent deformation of the bilayer, followed by rupture and complete coverage of the MSN particle.[3] Created with BioRender.com.

Characterisation:

DLS

Using a Malvern Zetasizer Nano with a 532 nm laser as the light source, Dynamic Light Scattering (DLS) analysis was performed, enabling the hydrodynamic size and ζ-potential of the vesicles to be obtained.

MRI

T_1 measurements were performed using an inversion recovery pulse sequence on a Spinsolve 60 Magritek 1.4 T NMR machine (2 scans, acquisition time 1.6 s, repetition time of 10 s, max inversion time of 5 s and 11 steps). The measurements were recorded for 0.5x, 0.25x and 0.125x dilutions of each sample (as previously described), with the r_1 values calculated from the gradient of the linear fit of a plot of $1/T_1$ versus the concentration of Gd (III) (as determined by ICP-MS).

ICP-MS

The Gd(III)-concentrations for each sample were calculated and verified using inductively coupled plasma mass spectrometry (ICP-MS) analysis (Perkin Elmer NexION 2000B). The samples were prepared by hydrolysing in 3 mL HNO_3 (70%) overnight. The samples were then adjusted to a final dilution of 2% HNO_3 using 18.2 MΩ deionised water. Solutions of a known Gd(III)-concentrations were employed for the external calibration process.

TEM

Transmission electron microscopy measurements were obtained employing a FEI Tecnai-12 (USA) microscope, operated at 120 kV. The samples were prepared by depositing a drop of an aqueous colloidal suspension of the liposomes, at a concentration of ~ 1 mg mL^{-1}, onto a copper grid. This was then negatively stained by incubating the grids in a 2% uranyl acetate stain, for 10 s. In the lysis studies of the liposomes, 20 µL of Triton™ X-100 detergent was used to lyse the phospholipid bilayer with subsequent release of encapsulated Gd-DOTA and small surfactant micelles.

Gd-DOTA-MSN characterisation

Double delay co-condensation NH_2-MSNs display a ζ-potential measurement of 14.5 ± 0.436 mV, a function of the high amination percentage of the NPs. Functionalisation with the negatively charged DOTA reduces the density of free NH_2-anchor points reduces the ζ-potential to 1.59 ± 0.116 mV. The

ζ-potential changes predictably upon Gd^{3+} chelation with the Gd-MSNs possessing an associated ζ-potential of -17.8 ± 0.404.

CTAB template removal is confirmed by ATR-IR, as shown in ESI 7. In order to quantify the density of chelated Gd^{3+} within the MSNs, inductively coupled plasma mass spectrometry (ICP-MS) analysis took place with the number of Gd^{3+} per particle calculated as per below:[4]

$$\frac{xM_{Gd}}{(M_{MSN} + xM_{GdDOTA})} = m\%_{Gd}$$

x = number of Gd^{3+} atoms per MSN, M_{Gd} = Mr of Gd, M_{MSN} = molar mass of silica scaffold, M_{GdDOTA} = Mr Gd-DOTA complex and $m\%_{Gd}$ = mass % of chelated Gd^{3+} (calculated by ICP-MS). Solving the for x estimates that the number of Gd^{3+} atoms per MSN is ~ 2700. (ESI 8).

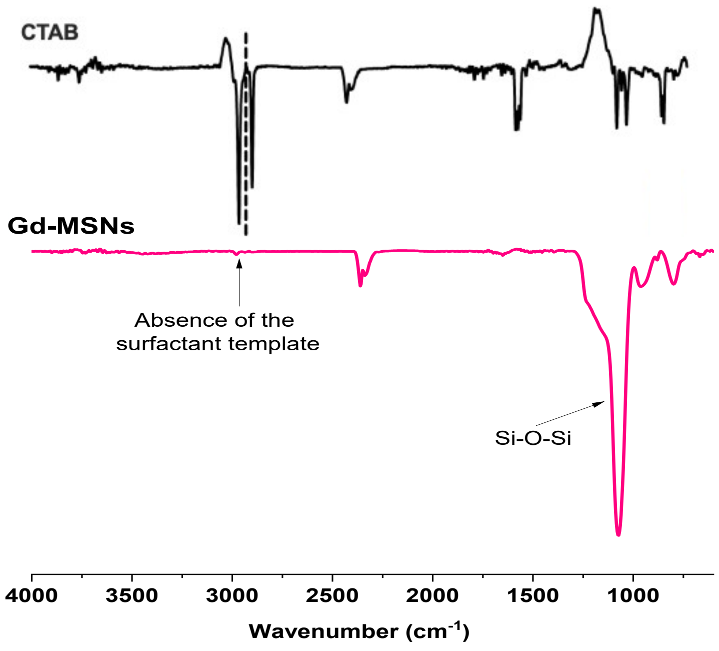

ESI 7. Attenuated total reflectance infrared spectroscopy (ATR-IR) measurements for the CTAB surfactant template (black) and Gd-DOTA-MSNs (pink). Here, both the Si-O-Si architecture and the removal of the template can be clearly observed.

Sample	Take (mg)	Dilute (mL)	c (mg/mL)	c ICP (mg/L)	c ICP (mg/mL)	wt %	wt%/100	Gd3+ / MSN

**NH_2-Gd-MSNs**	0.803	50	0.0160	0.115	0.000115	0.718	0.00718	2690

ESI 8. ICP-MS data from which Gd^{3+} per MSN is calculated for the NH_2-Gd-MSN architecture.

Clinical MR imaging was obtained, using the same methodology as described in our previous work.[5] Clinical images were obtained from scanners routinely used for clinical research: specifically, Siemens 3 T Prisma and Siemens Avanto Fit 1.5 T systems, both sited in a temperature-controlled, remotely monitored room. The samples were placed on the patient table with two large quality control fluid phantoms placed nearby for coil loading purposes: all imaging was undertaken using the body coil for RF transmit, and the spine array and 18-channel coils for RF receive on each scanner. After the acquisition of localisers and an automated shimming routine, shortened modified Look-Locker imaging (ShMOLLI) maps were acquired as described previously,[6] with a 192 x 144 acquisition matrix, 384 x 288 reconstruction matrix, 360 x 270 mm^2 FOV, 8 mm slice thickness, TE = 1.01 ms, TR = 2.05 ms, 35° readout flip angle, GRAPPA acceleration factor of 2 with 24 reference lines, 6/8 partial Fourier, inversion times 100 ms, 1100 ms, 2100 ms, 3100 ms, 4100 ms, 180 ms, 260 ms.

For the acquisition of Phantom MRI images (and associated T_1 values), the liposomal samples were prepared as follows:

Table 1. Sample preparation used for Phantom MRI studies.

**POPC-$C_{45\%}$-N**	**POPC-$C_{45\%}$-T**
800 µL 45 mol% cholesterol modified 75 mM Gd-DOTA doped POPC liposomes	800 µL 45 mol% cholesterol modified 75 mM Gd-DOTA doped POPC liposomes
7200 µL water	6960 µL water
	20 µL 1.25 mM Vln
	20 µL 1.25 mM chloride transporter
	200 µL KCl salt solution (10 mM, to give final KCl concentration of 1.08 mM in total solution)

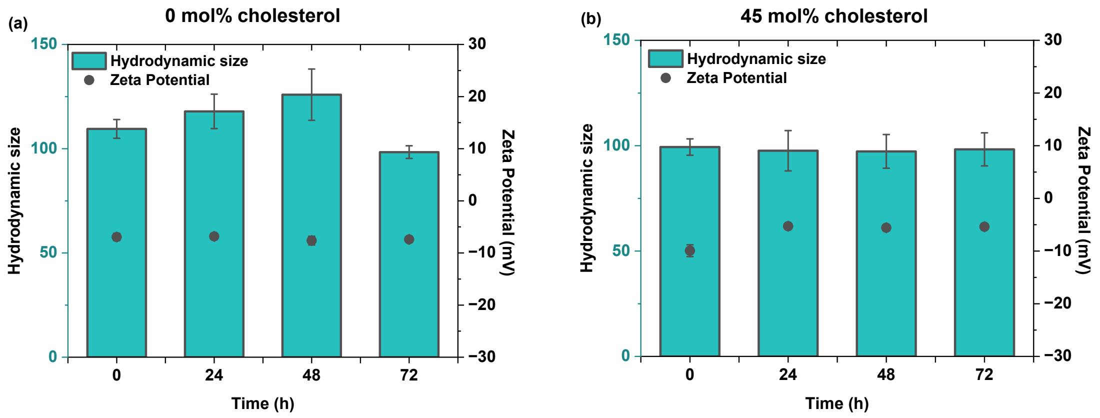

ESI 9. Stability measurements, recorded by DLS, for Gd-DOTA loaded POPC liposomes dispersed in water across 72 h. The results demonstrate the high colloidal stability of the vesicles over three days, with minimal variations in both the hydrodynamic size or ζ-potential for both the (a) 0 mol% and (b) 45 mol% cholesterol doped POPC liposomes.

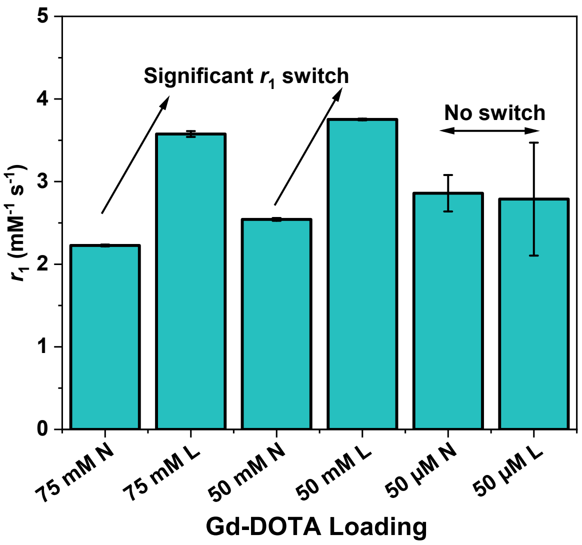

ESI 10. The longitudinal relaxivity values (obtained at 1.41 T, 298 K) calculated for both a native, 'N', and lysed (using 20 μL Triton), 'L', solution of the Gd-DOTA doped POPC liposomes (45 mol% cholesterol loading). The results are displayed for liposomes incorporating 75 mM, 50 mM and 50 μM of Gd-DOTA added at the point of synthesis, with the error bars referring to 1 s.d. of the mean value. There are no significant changes in relaxivity observed upon lysis for the liposomes containing 50 μM of Gd-DOTA due to the exceedingly low doping levels.

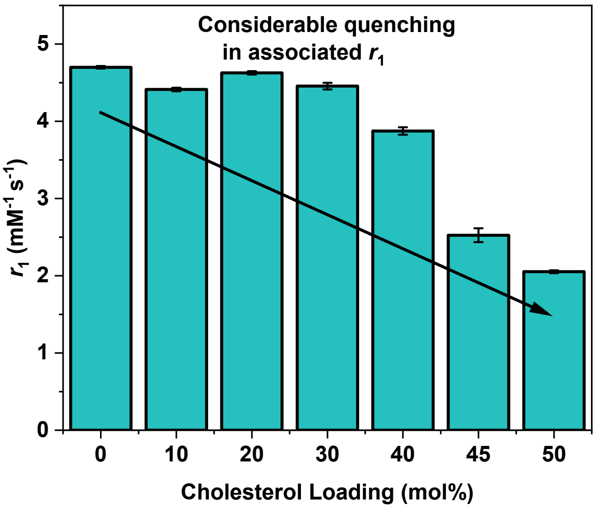

ESI 11. The longitudinal relaxivity values (obtained at 298 K, 1.41 T) for POPC liposomes containing a range of cholesterol dopant levels, varying from 0 mol% to 50 mol%. It can be observed that there is a significant reduction in r_1 as cholesterol doping density is increased (consistent with the presence of a rigidified bilayer), with a percentage decrease equal to 56.3% from the 0 mol% to the 50 mol% vesicles.

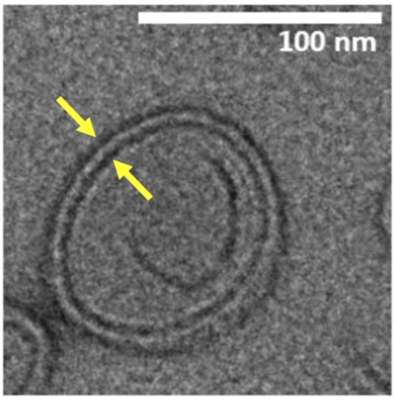

ESI 12. TEM image for the 45 mol% cholesterol doped POPC liposomes (chosen since this sample was employed for the transporter doped relaxivity analyses), possessing an associated diameter of 98.2 nm. Uranyl acetate was employed as a negative staining agent.

Elucidating the Stability of Liposomes in Serum

For POPC-$C_{45\%}$-N and POPC-$C_{45\%}$-T liposomes in 1.08 mM KCl (the predominant salt concentration used) stability measurements were carried out by DLS in the presence of 1%, 2% and 50% serum respectively. For ‘50% serum’ these samples were prepared by taking 0.5 mL liposomal solution in 1.08 mM KCl (POPC-$C_{45\%}$-N and POPC-$C_{45\%}$-T, separately) and 0.5 mL 100% serum in an Eppendorf tube. The sample tube was mounted on an analogue disk rotator for 2 hours, then analysed by DLS. This sample was then placed back in the rotator, and analysed again after 20 hours, and 90 hours.

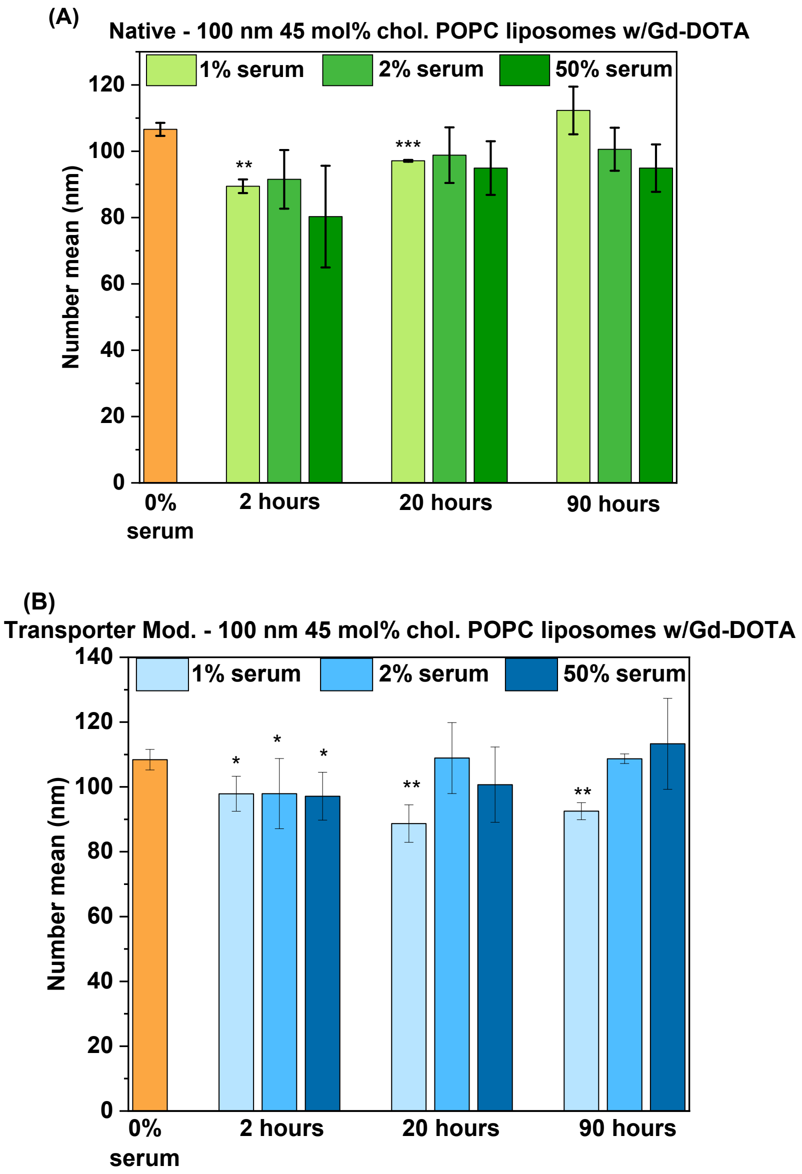

ESI 13. DLS analysis of POPC-$C_{45\%}$-N (A) and POPC-$C_{45\%}$-T (B) liposomes in 1.08 mM KCl in 1%, 2% and 50% serum respectively, reveals consistent hydrodynamic size with no overall trend (within error) over a time period of 90 hours, confirming robustness under physiological conditions. For both (A) and (B) the data points labelled with asterisks (* = $p < 0.05$, ** = $p < 0.01$, *** = $p < 0.001$) show statistically significant departure from the value in 0% serum (one-sample t-test). All other data points show departures from the mean which are not significant (n.s.). The different colour schemes are used to highlight the different liposome compositions present - POPC-$C_{45\%}$-N (a) and POPC-$C_{45\%}$-T (b).

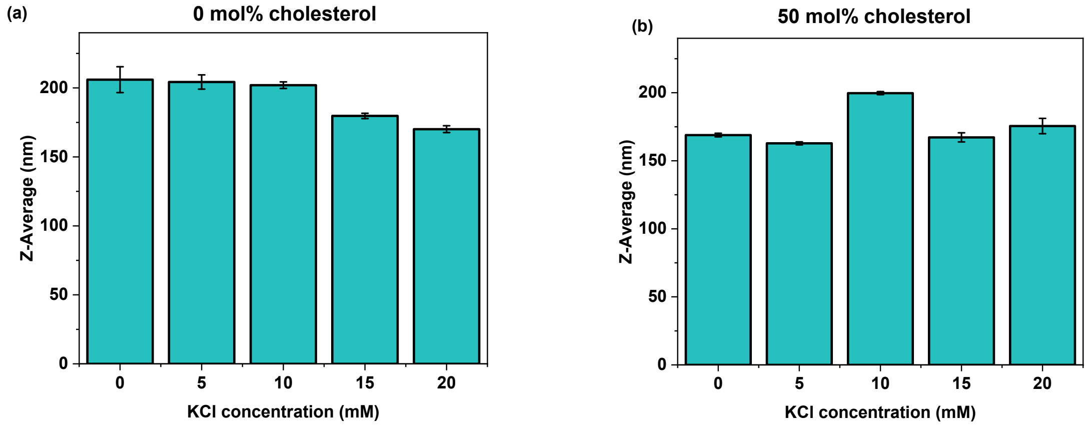

ESI 14. The average hydrodynamic size with an increasing osmotic imbalance (obtained through addition of a KCl salt solution, 0 – 20 mM) between the external bulk solution and the internal water pool of the liposome. (a) 0 mol% cholesterol doped liposomes. (b) 50 mol% cholesterol doped liposomes. For the 0 mol% liposomes there is a decrease in hydrodynamic size when comparing the results on addition of a low (0 mM) and high (20 mM) KCl solution, which is not observed for the 50 mol% liposomes, likely due to the presence of the rigidified bilayer.

End-Point Hydrodynamic Size Analysis by DLS

DLS data obtained for vesicles (ESI 18) containing KCl (300 mM buffered to pH 7.2 using HEPES 10 mM) were suspended in KGlc, potassium gluconate, (300 mM buffered to pH 7.2 using HEPES 10 mM) to make a test solution of 0.4 mM lipid in 0.8 mL external solution. A DMSO solution of either and/or both tripodal thiourea (4 µM, 1 mol%) and Valinomycin (0.4 µM, 0.1 mol%) was added, with the Z-average size (in nm) and ζ-potential (in mV) recorded after 30 minutes. These analyses demonstrate that hydrodynamic size is much more significantly reduced due to carrier-mediated KCl efflux for those liposomes with both transporters added, compared with those vesicle samples where either 0.1 mol% Vln *or* 1 mol% tripodal thiourea is added.

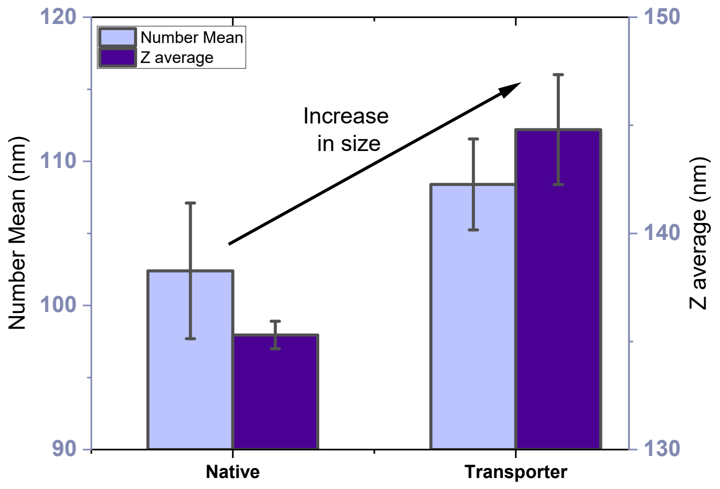

ESI 15. DLS measurements for Native and Transporter modified Gd-DOTA loaded POPC liposomes dispersed in 1.08 mM KCl. Observations demonstrate that the addition of anion and cation mobile ion carriers and resultant symport transfer of cations/anions across the lipid bilayer is associated with a swelling of the liposomal structure, as osmotic influx occurs, from a number mean of 102.4 ± 4.7 nm to 108.4 ± 3.1 nm, with an associated Z-average increase from 135.3 ± 0.6 nm to 144.8 ± 2.5 nm. This is due to the concurrent transfer of water with anions/cations across the liposomal membrane.

Osmotic Efflux Assays

Osmotic efflux assays were used to independently assess water flux across the lipid bilayer associated with M^+/X^- cation/anion symport.[7] Specific ion flux mediated liposome dehydration and shrinkage are detected in a temporal manner, and deswelling of the liposomal architecture increases in 90° light scattering as measured using a fluorimeter. This phenomenon can be modelled using the Rayleigh-Gans-Debye (RGD) approximation of Lorenz-Mie theory (rather than Rayleigh scattering, used to describe the behaviour of particles with a diameter much smaller than the wavelength of light).[8-10]

A 2.5 mM suspension of 400 nm 100% POPC liposomes, internal solution 300 mM KCl, 10 mM HEPES, pH 7.20; external solution 300 mM KGlc, 10mM HEPES, pH 7.20, was diluted to 0.4 mM for

spectroscopic data analysis 0.352 mL liposomes, 1.848 mL KGlc buffer, 15 µL DMSO transporter stock solution). The DMSO stock solution was varied as follows, (i) pure DMSO, (ii) 0.1 mol% Vln (final concentration 0.4 µM), (iii) 1 mol% T (final concentration 4 µM) and (iv) 0.1 mol% Vln and 1 mol% T; to allow for symport vs uniport ion transfer to be studied. Immediately after this addition, the 90° light scattering intensity was continuously monitored at 600 nm.

Upon mobile anion transporter (tripodal thiourea) and cation transporter (valinomycin) assisted symport transfer of K^+ and Cl^- from the internal pool of the liposome to bulk solution (KCl efflux), water efflux dissipates the osmotic imbalance generated by this ion flux. Osmotically induced reduction in liposome diameter (shrinkage) results in an increased refractive index (and membrane density), measured as an increase in light-scattering.[11-13]

The fractional light scattering intensity (I_t) was calculated using the following equation for the osmotic flux assay:

$$I_f = \frac{I_t}{I_0} - 1$$

$I_t = light\ scattering\ at\ time\ t, I_0 = light\ scattering\ when\ t = 0s$

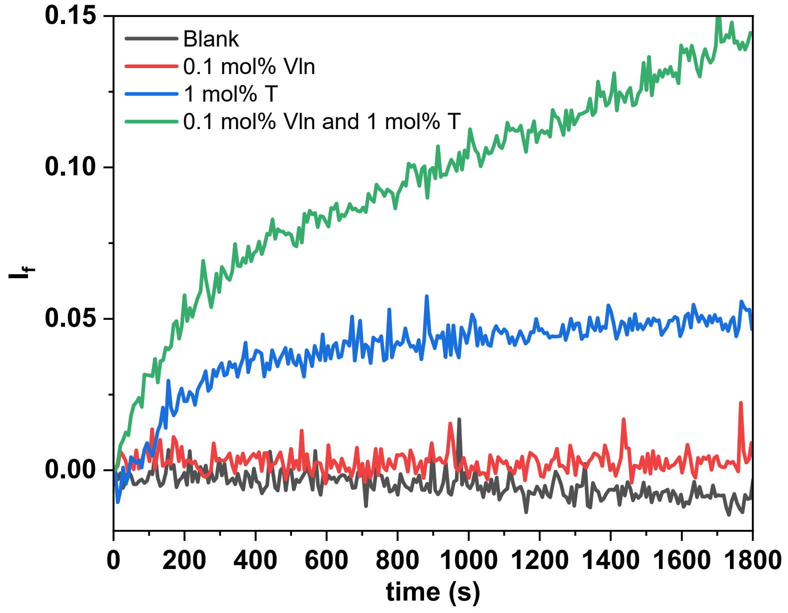

ESI 16. 90° light scattering, tracking average intensity increase upon cation and anion transporter addition to 400 nm POPC liposomes (internal 300 mM KCl, 10 mM HEPES, pH = 7.2, external 300 mM KGlc, 10 mM HEPES, pH = 7.2, measured at a wavelength of 600 nm). When symport transfer is

activated (upon dual integration of 0.1 mol% Vln and 1 mol% tripodal thiourea), the greatest enhancement in I_f is observed indicating significant enhancement in water efflux and vesicle shrinkage. A smaller response is observed for the sole addition of 1 mol% tripodal thiourea, likely due to Cl^-/OH^- antiport or H^+/Cl^- symport mechanisms.[7] As expected, negligible change in I_f is observed for either the blank (DMSO addition) or 0.1 mol% Vln. This is because, in the former, no transporters are present, and in the latter, there is no symport activation (no tripodal thiourea chloride transporter present) nor a concentration gradient to dissipate (300 mM K^+ in both internal liposomal pool and external bulk solution).

Water Flux Analysis by DLS

A 2.5 mM suspension of 400 nm 100% POPC liposomes, internal solution 300 mM KCl, 10 mM HEPES, pH 7.20; external solution 300 mM KGlc, 10mM HEPES, pH 7.20, was diluted to 0.4 mM for DLS analysis (0.128 mL vesicle suspension, 0.672 mL 300 mM KGlc, 10 mM HEPES, pH 7.20). The Z average of the liposomes was measured initially, prior to DMSO stock solution addition (time, t = 0 minutes). Following this, four different runs were trialled. The different experiments refer to: (i) a blank, (ii) cation transporter *only*, (iii) anion transporter *only*, and (iv) both cation *and* anion transporter. Therefore, individually, 5.45 µL of (i) pure DMSO (ii) 0.1 mol% Vln (final concentration 0.4 µM for DLS analysis), (iii) 1 mol% T (final concentration 4 µM) and (iv) 0.1 mol% Vln and 1 mol% T; were added, respectively, to allow for symport vs uniport ion transfer to be studied, commencing with end point analysis, as shown in ESI 17.

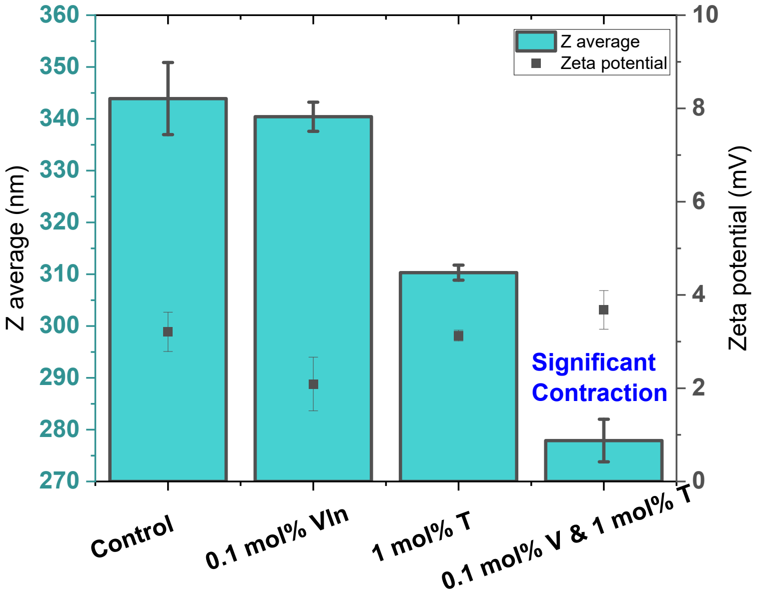

ESI 17. DLS measurements displaying the concurrent reduction in hydrodynamic diameter associated with ion transporter mediated ion exchange of KCl from the internal pool to the bulk solution (H_2O efflux).

From this, time dependence of size and thus liposomal volume and associated surface area could be tracked at repetitive time intervals over the course of the deswelling process. This was conducted via continuous DLS measurements of Z average during > 1 hour, as shown in ESI 18.

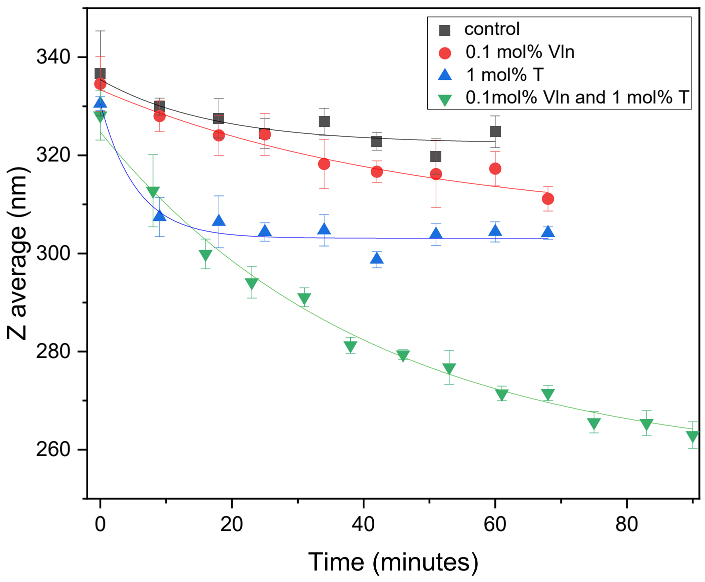

ESI 18. DLS measurements displaying the concurrent reduction in hydrodynamic diameter associated with ion transporter mediated ion exchange of KCl from the internal pool to the bulk solution and associated H_2O efflux.

From this analysis, assuming (as standard) that the liposomal population is homogenous and spherical, the change in volume (and surface area) over time can be elucidated.[14-16] Hydrodynamic size measured by DLS (ESI 18) was converted to a volume using $^4/_3.\pi.(^d/_2)^2$, and a surface area, $\pi.d^2$. From the plot of liposomal volume vs time. This gives a volume function measured in nm^3 /s , which is tracked over time. Taking the volume occupied by one H_2O molecule to be 2.99 x 10^{-23} cm^3 (33.3 per nm^3),[17] the number of water molecules / s^2 was calculated using -33.3 x volume'(s). From a plot of liposomal membrane surface area vs time, either a double exponential decay function is fitted (0.1 mol% Vln and 1 mol% Tripodal thiourea) or a single exponential decay function is fitted (control – DMSO blank added). This gives a surface function measured in nm^2 /s . Finally, the water flux is calculated by dividing the rate of change of water molecules by the surface function, to give a net water efflux measurement, in units of number of water molecules $nm^{-2}s^{-1}$.

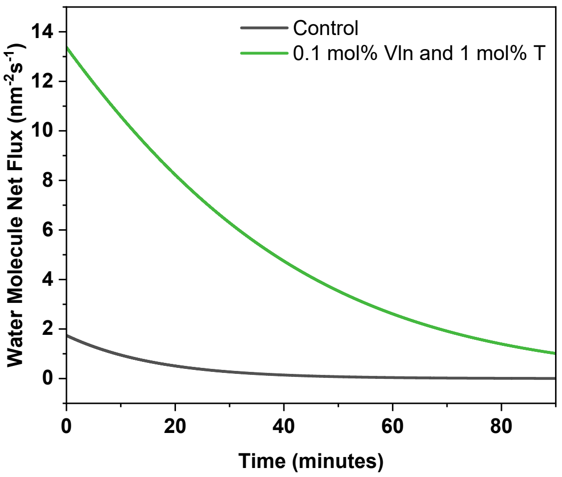

ESI 19. Plot of the number of water molecules per nm^2 per second transferred across the membrane vs time (minutes) calculated directly from the DLS data (ESI 18) which tracks the decrease in volume in real-time associated with ion transporter mediated ion efflux of KCl for the control and 0.1 mol% Vln and 1 mol% tripodal thiourea sample. Here, the addition of ionophores greatly elevates the water flux rate across the liposomal membrane, compared with a control (un-modified) lipid bilayer.

Table 2. Representative cross comparison calculations to validate/corroborate the analyses as performed in ESI 19, for the final (deswelling at 90 minutes) number of water molecules undergoing efflux per second per nm^2 (both transporters integrated).

Initial Diameter of Liposome(nm)	328
Final Diameter of Liposome (nm)	263
Volume of one water molecule (nm^3)	0.03
Time of Experiment (s)	5400
Initial Volume of Liposome (nm^3)	1.48E+08
Final Volume of Liposome (nm^3)	76200000
Difference in volume (nm^3)	71800000
Number of water molecules that have undergone efflux (molecules)	2.39E+09
Water molecule efflux per second (molecules s^{-1})	443000
Average diameter of liposome (nm)	296
Average Surface area of one liposome (nm^2)	274000
Water molecule efflux per second per unit area (molecules s^{-1} nm^{-2})	1.62

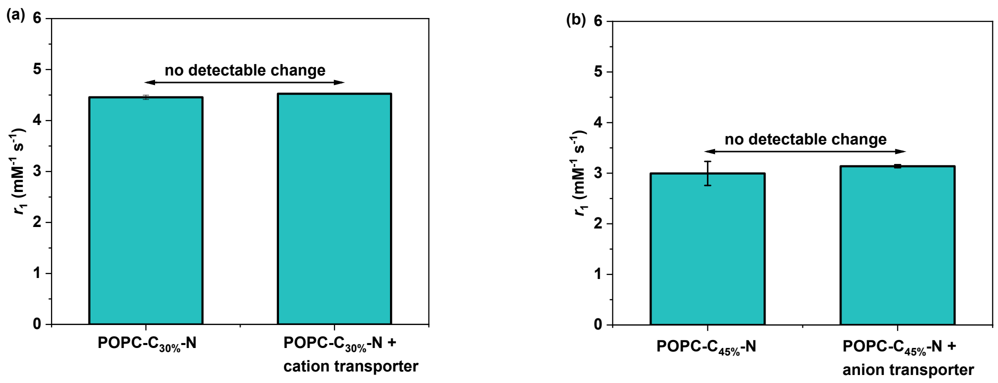

ESI 20. No detectable change in longitudinal relaxivity at 1.41 T (and 298 K) is obtained upon addition of a 0.5 mL 10 mM KCl solution to (a) 1 mol% (w.r.t. POPC) Valinomycin DMSO stock solution ***alone*** in POPC-C$_{30\%}$-N liposomes, and (b) 1 mol% (w.r.t. POPC) tripodal thiourea DMSO stock solution ***alone*** in POPC-C$_{45\%}$-N liposomes.

Lucigenin Ion Transport Assay[18,19]

To prepare the liposomes used for the lucigenin transport assay, 500 µL 25 mg mL^{-1} POPC stock solution in $CHCl_3$ was transferred to a clean and dry 10 mL RBF. The solvent was removed using rotary evaporation to form a lipid film, followed by drying under high vacuum for 6 h. To this thin film, 1.0 mL of 112.5 mM K_2SO_4, 5 mM HEPES, 1.0 mM lucigenin aqueous solution was added (pH 7.2). Vortexing was used to fully suspend the lipids in solution. This suspension then underwent 5 freeze-thaw cycles, using liquid nitrogen and a 30 °C water bath, before being extruded 19 times through a 200 nm pore size polycarbonate membrane. Using Sephadex G-50, size exclusion chromatography was performed, using 112.5 mM K_2SO_4 5 mM HEPES as the eluent, to remove any non-encapsulated lucigenin. The vesicle solution was diluted to 2 mL, giving a final solution concentration of 5 mM 200 nm 100% POPC vesicles, interior 112.5 mM K_2SO_4, 5 mM HEPES, 1.0 mM lucigenin; exterior 112.5 mM K2SO4 5 mM HEPES, pH 7.2 interior and exterior.

In a clean and dry fluorescence cuvette, 112.5 mM K_2SO_4, 5 mM HEPES (2880 µL), prior-prepared 200 nm POPC vesicles (40 µL, 109.6 µM) and 0.1 mol% valinomycin (20 µL in MeOH solution) and/or 1

mol% tripodal thiourea (20 µL in THF solution) were added. This cuvette was placed in a Agilent Cary Eclipse fluorescence spectrophotometer equipped with a magnetic stirrer. The fluorescence intensity of lucigenin was monitored at I_{em} = 535 nm (I_{ex} = 455 nm) over time. The chloride gradient was created by a 2.0 M KCl (40 µL) pulse and the change in I_f tracked. Afterwards, the vesicles were lysed by adding 10% Triton X-100 (40 µL) to dissipate the chloride gradient. To normalise the time-dependent data to the relative change in fluorescence intensity, the following equation is used:

$$I_f = \left(\frac{I_t}{I_0}\right)$$

Where I_0 is the initial intensity and I_t is the intensity at time t. The lucigenin assay data is displayed in ESI 21.

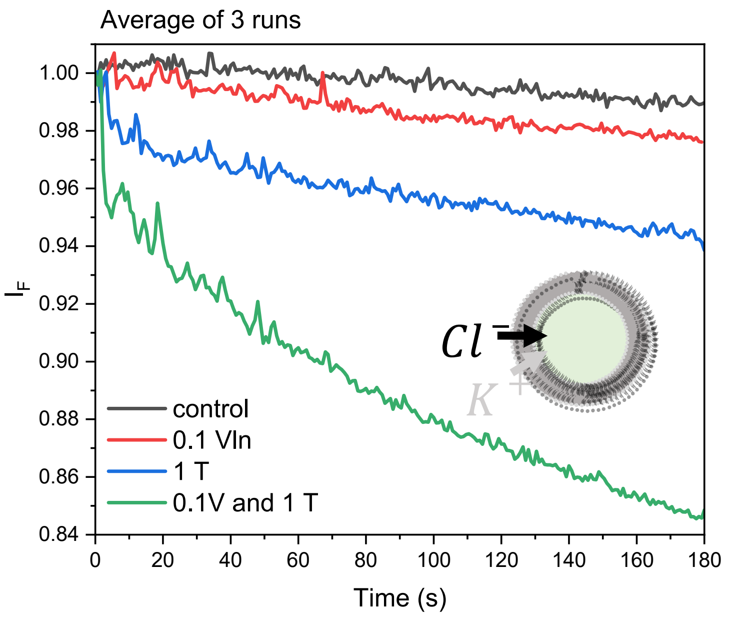

ESI 21. Ion transport determined using the lucigenin. which tracks KCl symport from bulk solution by both anion and cation mobile ion carriers through the liposomal membrane, resulting in a quenching of internal lucigenin (l_{em} = 535 nm ,l_{ex} = 455 nm). The relative change in fluorescence intensity, induced by chloride quenching of the encapsulated fluorophore upon KCl transport, is shown for four samples: control, 0.1 mol% valinomycin (0.1 Vln), 1 mol% tripodal thiourea (1 T) and both 0.1 mol% valinomycin *and* 1 mol% tripodal thiourea (0.1V and 1 T) (averaged over three independent runs). Both the initial drop due to quenching of external lucigenin and the horizontal plateau (before KCl addition) have been removed for reasons of clarity.[19]

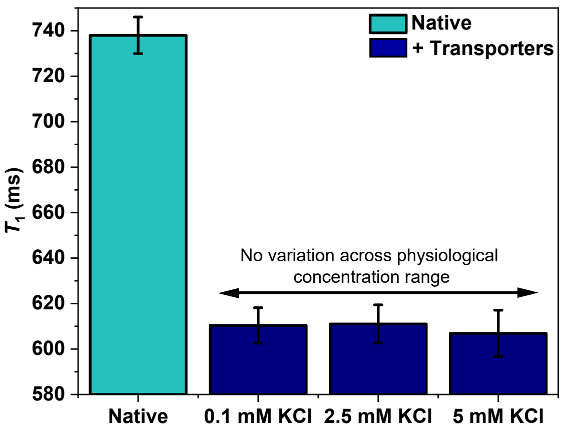

ESI 22. T_1 analysis, comparing POPC-$C_{45\%}$-N with POPC-$C_{45\%}$-T suspended in a variety of KCl concentrations across the physiological concentration range, spanning 0.1 mM to 5 mM. No detectable change in T_1 at 1.41 T (and 298 K) is obtained upon varying the external concentration of KCl solution added to POPC-$C_{45\%}$-N liposomes, to give a final concentration of KCl in solution of 0.1 mM, 2.5 mM and 5 mM KCl respectively (consistent total volume across samples to allow for accurate data comparison).

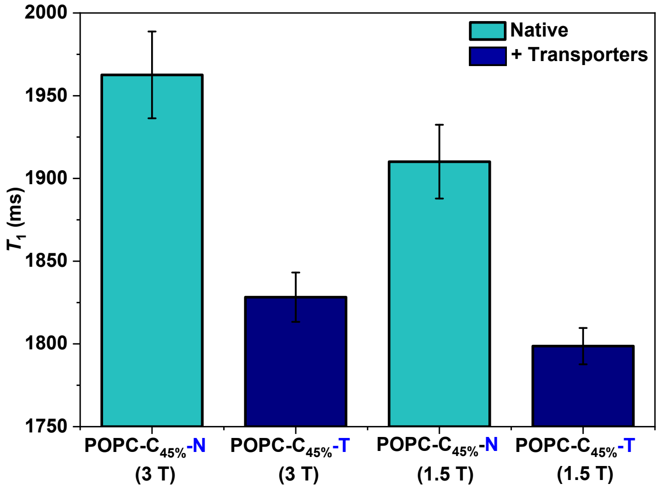

ESI 23. The associated MRI relaxation times for the Gd-doped 45 mol% cholesterol liposomes, recorded at 3 T and 1.5 T (298K) This clinical data was acquired for POPC-$C_{45\%}$-T nanoparticles in the presence of KCl salt (100 µL of 0.1 mM solution) employing a 1 mol% valinomycin solution and 1 mol% tripodal thiourea solution. The table above displays the components of such formulations used within the clinical MRI scanner.

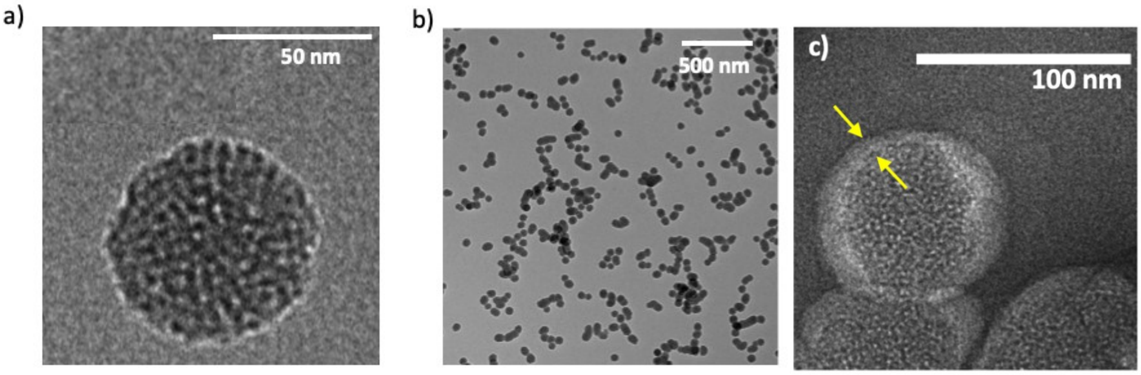

ESI 24. Transmission electron microscopy (TEM) images of a), b), Gd-MSNs and c) POPC-$C_{0\%}$-MSN. The scale bar corresponds to a) 50 nm, b) 500 nm and c) 100 nm respectively. (c) demonstrates complete wrapping of the MSN architecture by a POPC lipid bilayer.

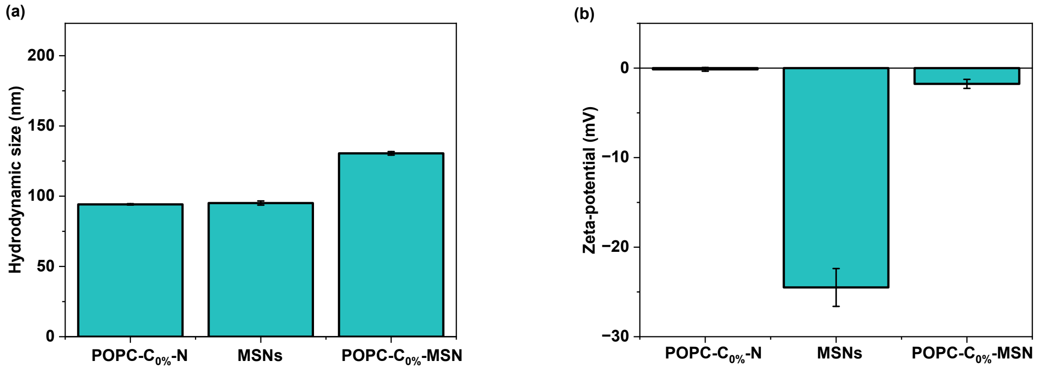

ESI 25. (a) Hydrodynamic size (nm) and (b) ζ-potential (mV) measurements, recorded by DLS, for the liposomes (0 mol% cholesterol loading), MSNs and POPC-$C_{0\%}$-MSNs.

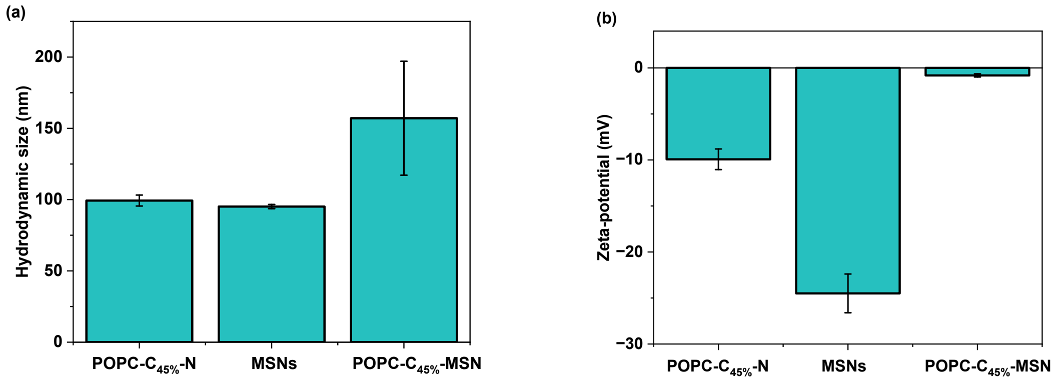

ESI 26. (a) Hydrodynamic size (nm) and (b) ζ-potential (mV) measurements, recorded by DLS, for the liposomes (45 mol% cholesterol loading), MSNs and POPC-$C_{45\%}$-MSNs demonstrating complete wrapping of the MSNs.

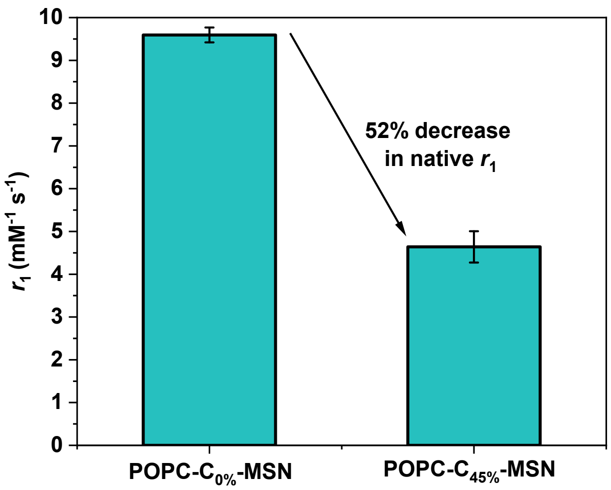

ESI 27. Longitudinal relaxivity values (obtained at 1.41 T, 298K) for the native POPC-$C_{0\%}$-MSN and POPC-$C_{45\%}$-MSN nanoparticles (in the absence of the ionophores). A relaxivity dampening of 52% on rigidification of the bilayer with cholesterol is highlighted. This is consistent with the results observed for the Gd-DOTA doped liposomes, which exhibited a 46% decrease in r_1 between 0 and 45 mol% vesicles.

References:

1. J. J. Davis *et al., J. Mater. Chem.,* 2012, **22**, 22848-22850.
2. K.S. Butler *et al., Small*, 2016, **12**, 2173–2185.
3. O. Le Bihan *et al.*, *J. Struct. Biol.*, 2009, **168**, 419–425.
4. W. J. Rieter *et al.*, *Angew. Chem. Int. Ed.*, 2007, **46**, 3680–3682.
5. C.M. Ellis, *et al., Chem Commun.*, 2023, **59**, 1605–1608.
6. S.K. Piechnik *et al., J. Cardiovasc. Magn. Reson.,* 2010, **12**, 69.
7. H. J. Clarke *et al., J. Am. Chem. Soc.,* 2016, **138**, 16515–16522.
8. J. H. Van Zanten and H.G. Monbouquette, *J. Colloid Interface Sci., 1994, **165**,* 512–518.
9. P.J. Wyatt, *Anal. Chem.*, 2014, **86**, 7171–7183.
10. R.Y. Ofoli and D.C. Prieve, *Langmuir*, 1997, **13**, 4837–4842.
11. R. Bittman and L. Blau, *J. Chem. Educ.,* 1976, **53**, 259.
12. T.P. Silverstein and J.C. Williamson, *Biochem. Mol. Bio. Educ,* 2019, **47**, 239–246.
13. L.I. Viera *et al.*, *Chem. Phys. Lipids*, 1996, **81**, 45–54.
14. X. Xu, *et al.*, *J. Pharm.*, 2012, **423**, 410–418.
15. K. Matsuzaki *et al., Biochim. Biophys. Acta (BBA) – Biomembr.*, 2000, **1467**, 219–226.
16. L. Cliff *et al., Biochim. Biophys. Acta (BBA) – Biomembr.*, 2020, **1862**, 183033.
17. R. Araya-Secchi *et al., BMC Genom.*, 2011, **12**, S8.
18. M Ahmad *et al., Angew. Chem. Int. Ed.*, 2024, **63**, e202403314.
19. M. Chvojka *et al., Analysis & Sensing,* 2024, **4**, e202300044.
